# Comparison of the Sedative Effects of Dexmedetomidine and Remifentanil in Vitrectomy Surgery: A Retrospective Study

**DOI:** 10.7759/cureus.46204

**Published:** 2023-09-29

**Authors:** Tuba Kuvvet Yoldaş, Nevin Esra Gümüş

**Affiliations:** 1 Clinic of Anesthesiology and Reanimation, University of Health Sciences Turkey, Tepecik Training and Research Hospital, İzmir, TUR; 2 Clinic of Anesthesiology and Reanimation, University of Health Sciences Turkey, Samsun Education and Research Hospital, Samsun, TUR

**Keywords:** conscious sedation, anaesthesia, remifentanil, dexmedetomidine, vitrectomy

## Abstract

Background

Vitrectomy surgery is a painful and lengthy procedure. Therefore, administering sedation to reduce patient agitation provides both surgical comfort and hemodynamic stability. However, various complications can arise during the perioperative period depending on the sedation agent used. In our study, we aimed to evaluate the effects of dexmedetomidine and remifentanil sedation applications on patient hemodynamics and perioperative complications in vitrectomy surgery.

Methods

Our retrospective study included patients aged 18-70 who underwent vitrectomy surgery between 2021 and 2022 with complete file data and ASA scores of 1-3 after obtaining approval from our hospital's ethics committee. Patients were classified into two groups based on the sedation agent used: Group D for dexmedetomidine and Group R for remifentanil. Demographic data of patients, heart rate, mean arterial pressure, oxygen saturation, and bispectral index values during perioperative monitoring, operation duration, and complications such as perioperative nausea, vomiting, and low saturation were recorded. The data of both groups were statistically evaluated, with p<0.05 values considered statistically significant.

Results

Because of missing data in 18 out of 58 patient files, these cases were excluded from the study. A total of 40 patients were included in the study, with 20 in Group D and 20 in Group R. The mean age of the patients was 64. Among them, 18 (45%) were male, and 22 (55%) were female. The mean operation duration was 61.8 ± 24.1 minutes in Group D and 56.3 ± 17.2 minutes in Group R. The heart rate in Group D was statistically significantly lower than in Group R, starting from the 20th minute of the perioperative period. There were no significant differences between the groups in terms of mean arterial pressure, oxygen saturation, and bispectral index values. One case of bradycardia occurred in Group D, requiring intervention.

Conclusion

In vitrectomy surgery, both dexmedetomidine and remifentanil infusions can be used for sedation, but caution is advised regarding bradycardia in Group D. Anticipating potential complications with an experienced anesthesia team is crucial for both patient and surgical comfort.

## Introduction

In ocular surgery, local anesthesia or regional anesthesia is generally preferred. However, even with these anesthesia techniques, issues such as pain, fear, and anxiety can still arise [1-2]. Vitrectomy surgery can be performed with peribulbar anesthesia and intravenous (IV) sedation support, allowing the procedure to be completed in most patients without the need for general anesthesia [3]. Given that vitrectomy surgery is a painful and lengthy procedure; therefore, sedation is applied to reduce patient agitation, ensuring both surgical comfort and hemodynamic stability.

In retina surgery, anesthetic agents such as propofol, midazolam, dexmedetomidine, and opioids are used for sedation purposes. These agents are preferred to mitigate the development of tachycardia and hypertension due to patient agitation and pain. However, the use of anesthetic agents can result in perioperative hemodynamic instability, including bradycardia and hypotension. Ensuring hemodynamic stability with reliable sedative drugs enhances surgical comfort. Additionally, these drugs can cause adverse respiratory complications, potentially hindering the patient's cooperation during surgery. We hope that the choice of sedative agents, such as dexmedetomidine and remifentanil, will provide superior hemodynamic control, ultimately leading to a safer surgical environment.

Among opioid agents, remifentanil is a μ-opioid receptor agonist. Different from other opioids, its effectiveness starts rapidly, and it has a short elimination half-life of 3-10 minutes. Because of these properties, remifentanil is often used either in combination with propofol or as a standalone agent for sedation [4]. On the other hand, dexmedetomidine is a potent, selective, and specific α2-adrenergic agonist with dose-dependent sedative and analgesic properties, without causing respiratory depression [5-6]. Dexmedetomidine is the preferred agent for sedation in retinal surgery because of its ability to maintain hemodynamic and respiratory stability [7].

In our clinical practice, we use two sedation agents in vitrectomy surgery. We wanted to evaluate our past experiences based on the data we have. Our goal is to understand the effectiveness of our practice with these results. Our retrospective study may also guide future prospective studies.

The primary aim of this study was to assess and compare the effects of dexmedetomidine and remifentanil sedation on patient hemodynamic parameters during vitrectomy surgery. The secondary objective of the study was to evaluate the incidence of perioperative complications associated with both dexmedetomidine and remifentanil.

## Materials and methods

In our retrospective study, patients aged 18-70 who underwent vitrectomy surgery between 2021 and 2022 had ASA scores of 1-3 and had complete medical data were included. The study was initiated with the approval of the Tepecik Education and Research Hospital Ethics Committee (Approval no: 2023/05-13). Patients with a history of cerebrovascular disease, severe respiratory or cardiovascular insufficiency, hemoglobin levels below 8 g/dL, and those who received general anesthesia were excluded from the study. Patient data were recorded from the hospital information system’s anesthesia forms.

In our routine practice, patients are subjected to topical anesthesia with a 0.5% proparacaine solution and then sedation is initiated with anesthetic agents. In ophthalmic surgery, both dexmedetomidine and remifentanil intravenous infusions are administered by an anesthesiologist. In our clinical practice, the dexmedetomidine infusion is administered at a dose of 0.25 mcg/kg over 5-10 minutes, then a maintenance infusion rate of 0.2-0.5 mcg/kg/min when the bispectral index (BIS) value is between 70 and 80. The remifentanil infusion is administered at a dose of 1 mcg/kg over 5-10 minutes, then a maintenance infusion rate of 0.1-0.3 mcg/kg/min when the BIS value is between 70 and 80.

The patients were divided into two groups according to the sedation agent used: Group D for dexmedetomidine and Group R for remifentanil. The demographic parameters (age, weight, ASA score), comorbidities, and operative parameters were recorded by reviewing the patient files. Heart rate (HR), mean arterial pressure (MAP), oxygen saturation (SpO2), and BIS values were measured at 0, 5, 10, 15, 30, and 60 minutes during the operation and the operation duration and complications such as perioperative nausea, vomiting, and SpO2≤90%, were recorded on the case report form. For primary outcomes, the hemodynamic changes between the two groups were compared. Additionally, complications between the two groups were compared.

The collected data were analyzed using the Statistical Product and Service Solutions (SPSS) (version 24; IBM SPSS Statistics for Windows, Armonk, NY). Descriptive statistics were used to present the distribution of responses to independent variables, with categorical variables presented as numbers and percentages, and numerical variables presented as means, standard deviations, and medians. The normal distribution of quantitative data was evaluated using the Shapiro-Wilk test. In the comparisons between groups, Student's t-test was used for data showing normal distribution, and the Mann-Whitney U test was used for data not showing normal distribution. For repeated measurements within each group, repeated measures ANOVA was used for normally distributed data, followed by the Bonferroni test for post-hoc analysis, and the Friedman test was used for non-normally distributed data, followed by the Dunn test for post-hoc analysis. Results were considered statistically significant, with a P value of ≤0.05.

## Results

**Figure 1:**
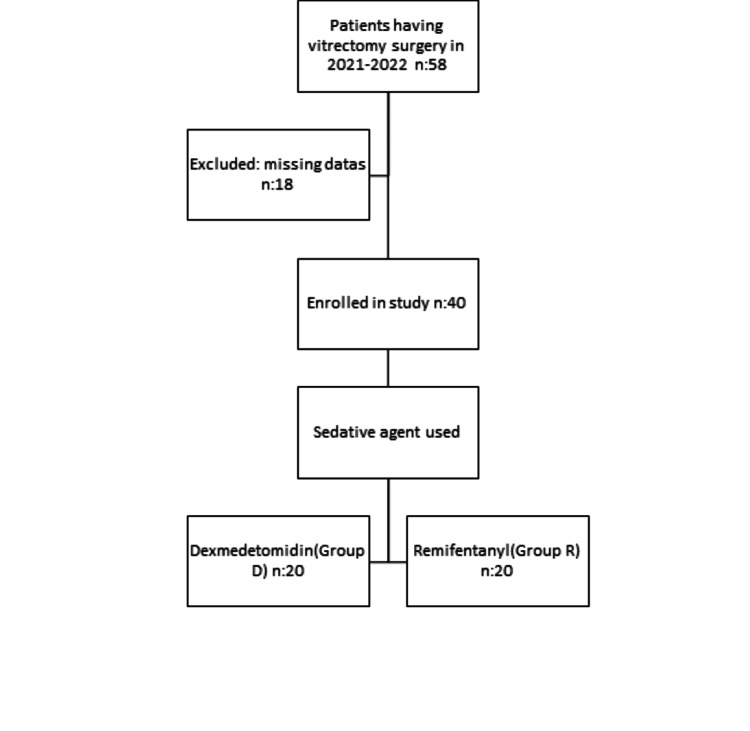
Participant flow diagram for the eligibility within the study

Fifty-eight patients who underwent vitrectomy surgery between 2021 and 2022 and were sedated with either remifentanil or dexmedetomidine, in addition to topical anesthesia, were included in the study. Eighteen out of 58 patient files were excluded due to missing data. A total of 40 patients, 20 in Group D and 20 in Group R, participated in the study (Figure 1). Furthermore, it was confirmed that there were no instances necessitating the initiation of sedoanalgesia with subsequent transition to general anesthesia throughout the follow-up period.

Patients’ mean age was 64 years. Of these, 18 (45%) were male, and 22 (55%) were female. There was no statistically significant difference between the groups in demographic data (Table 1).

**Table 1:** Patients’ demographic data and complications M: male; F: female; ASA: American Society of Anesthesiologists; group D: group dexmedetomidine; group R: group remifentanil

	Group D (n=20)	Group R (n=20)
Sex (M/F, n)	10/10	8/12
Age (years, mean ± SD)	63 ± 3.4	65 ± 2.7
Diseases in the medical history (n) No/Yes	6/14	5/15
ASA score (n) I/II/III	6/12/2	5/12/3
Operation time (min)	61.8 ± 24.1	56.3 ± 17.2
Complications (n) bradycardia/hypotension/hypoxia	1/0/0	0/0/0

When the regularly measured hemodynamic parameters of both groups were examined, it was observed that the HR in Group D was statistically significantly lower than in Group R from the 20th minute of the surgery onwards (Table 2). There were no significant differences observed between the groups in terms of mean blood pressure (MBP) and SpO2 values. BIS values began decreasing from the 15th to the 20th minute, but there was no significant difference between the two groups.

**Table 2:** Comparison of the HR values of the groups *p≤0.05; group D: group dexmedetomidine; group R: group remifentanil; HR: heart rate

Time (min)	Group D (mean ± SD)	Group R (mean ± SD)	p-value
Preop	73.7 ± 15.7	74.7 ± 9.5	0.818
After induction	69.7 ± 15.3	71.7 ± 7.5	0.594
15th	63.6 ± 12.5	69.3 ± 8.5	0.100
20th	61.9 ± 11.4	69.5 ± 8.8	0.023*
25th	61.4 ± 10.9	69 ± 6.8	0.012*
30th	59.9 ± 10	68.5 ± 8.5	0.006*
45th	58.6 ± 8.4	67.7 ± 8	0.003*
60th	61 ± 12.1	65.8 ± 3.5	0.202

**Figure 2:**
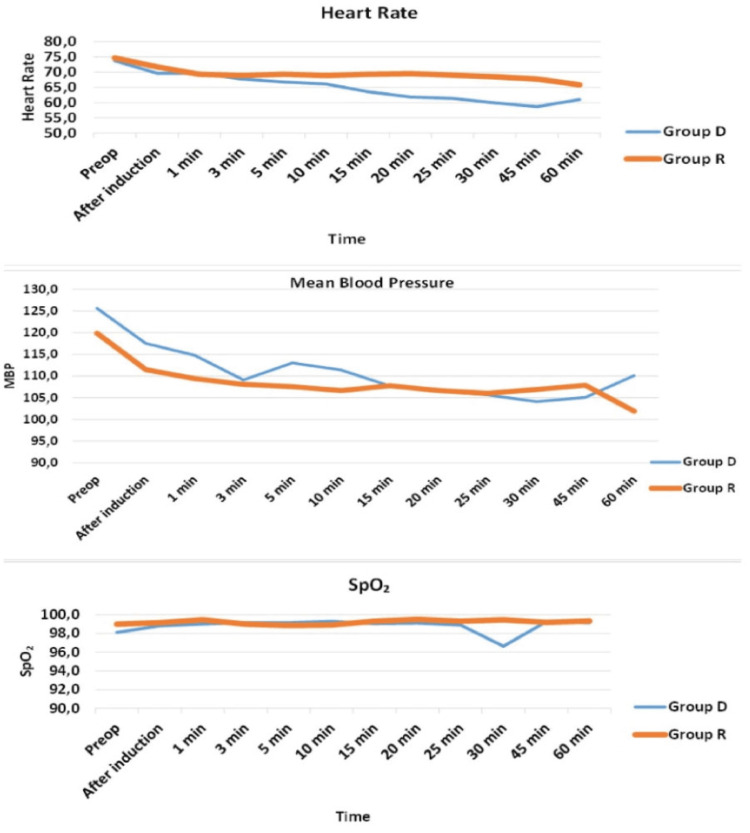
Comparison HR, MBP, and SpO₂ values between groups HR: heart rate; MBP: mean blood pressure; SpO₂: oxygen saturation; group D: group dexmedetomidine; group R: group remifentanil

The duration of surgery was 61.8 ± 24.1 minutes in Group D and 56.3 ± 17.2 min in Group R. There was no statistically significant difference in the average surgical duration. During the surgical procedure, in Group D, intervention with 0.5 mg IV atropine was performed in only one patient when their heart rate (HR) dropped below 50 beats/min. In Group R, no complications developed (Table 1). Hemodynamic values were observed to be more stable in Group R patients (Figure 2).

## Discussion

This study conducted on adult patients who underwent vitrectomy surgery demonstrates that both dexmedetomidine and remifentanil infusions are suitable for sedation in retinal surgery. However, it is important to exercise caution with dexmedetomidine infusion because of a higher risk of bradycardia compared to remifentanil infusion.

Sedation in ophthalmic surgery provides immobilization of patients, cooperation as needed with the patient, maintaining low to moderate intraocular pressure for ophthalmologists, ensuring a clean surgical field, and achieving the cardiovascular and respiratory stabilization desired by anesthetists [8]. Sedation enhances patient comfort, maintains low intraocular pressure, and prevents hypertensive responses [9].

Both dexmedetomidine and remifentanil are commonly used agents for conscious sedation in routine practice. Dexmedetomidine may have hemodynamic effects such as a decrease in blood pressure and bradycardia. However, in patients with a history of cardiovascular diseases such as hypertension, dexmedetomidine offers an advantage in maintaining hemodynamic stability during surgical stress [10]. Yoo et al.'s study in 2015 divided the patients into two groups: the control group, who received subtenon anesthesia only, and the dexmedetomidine group: who received dexmedetomidine in addition to subtenon anesthesia. Intravenous saline infusion was administered to the control group, and intravenous dexmedetomidine was administered to the dexmedetomidine group. In the dexmedetomidine group, blood pressure and heart rate were significantly lower than in the control group. A patient whose heart rate was below 40 beats/min was treated with atropine. Patient and surgeon satisfaction was higher in the dexmedetomidine group. Additionally, pain was less in the dexmedetomidine group than in the control group [11]. In our study, blood pressure remained stable in the dexmedetomidine group; however, bradycardia from 20 minutes onwards was significantly higher compared to the remifentanil group.

Remifentanil, due to its predictable short duration of action and strong analgesic effect, is preferred for sedation in the form of infusion. However, remifentanil can cause respiratory depression. Additionally, it can lead to hypotension and bradycardia, especially in elderly and frail patients [12]. Despite the relatively older age of our patient group, we did not encounter complications such as bradycardia, hypotension, or respiratory apnea. The patients in our study exhibited more stable hemodynamics under remifentanil infusion. In a study by Park et al. [13] when the dexmedetomidine-administered group (Group D) and remifentanil-administered group (Group R) were compared in terms of hemodynamics and respiration during cataract surgery, Group D exhibited higher heart rate and respiratory rate, but there was no difference in MAP and SpO₂. The study also highlighted that Group D had deeper anesthesia and lower surgeon satisfaction because of increased patient cooperation difficulty. In contrast, in our study, the stability of hemodynamics and absence of respiratory depression in Group R may be reasons for its preference.

The use of dexmedetomidine for retinal surgical procedures under topical anesthesia shows that it is effective in providing safe, awake sedation and analgesia. Abdalla et al. noted that sedation with dexmedetomidine in retinal surgery resulted in surgeon satisfaction and comfort, lower pain scores for patients, patient cooperation, and stable vital signs in the operating room and recovery room [9]. Remifentanil infusion also has an effective analgesic effect. However, pain, patient comfort, and surgical comfort could not be evaluated in our study.

A limitation of this study was that the sample size was limited. Additionally, since it is retrospective, patient and surgeon comfort cannot be evaluated. Retrospective studies inherently carry a predisposition towards bias. While conducting our study, we exercised utmost diligence in patient selection, data analysis, and processing. Nevertheless, it is important to acknowledge that, unlike randomized studies, we were unable to fully mitigate bias since our evaluators were not blinded. As a future avenue of investigation, we propose the validation of our findings through prospective randomized studies.

## Conclusions

Dexmedetomidine or remifentanil infusion can be used effectively and safely for sedation in vitrectomy surgery. Remifentanil sedation has less effect on hemodynamics during vitrectomy and provides hemodynamic stability. However, the hemodynamic effect is greater in dexmedetomidine sedation. More bradycardia is observed in patients using dexmedetomidine. In the future, more studies are needed. Comparing postoperative patient and surgeon satisfaction in future studies will also be useful in terms of the effectiveness of medications. Correct evaluation of the patient by an experienced anesthesia team and prediction of possible complications are very important for both patient comfort and surgical satisfaction.
